# Effects of a physical exercise program on HIF-1α in people with Chronic Obstructive Pulmonary Disease living at high altitude: study protocol for a clinical trial

**DOI:** 10.1186/s13063-023-07698-y

**Published:** 2023-10-29

**Authors:** Wilder Villamil-Parra, Édgar Cristancho-Mejía, Joan Ramon Torrella, Erica Mabel Mancera-Soto

**Affiliations:** 1 Department of Human Body Movement, Faculty of Medicine, Universidad Nacional de Colombia, Bogotá Campus, Street 30 No. 45-03 No. 45-03 Building 471, Bogotá, D.C. 110821 Colombia; 2 Department of Biology, Faculty of Sciences, Universidad Nacional de Colombia, Bogotá Campus, Street 30 No. 45-03, Bogotá, D.C. 110821 Colombia; 3 Physiology Section, Department of Cellular Biology, Physiology and Immunology, Faculty of Biology, Universitat de Barcelona, Avenue Diagonal, Barcelona, 643, 08028 Spain

**Keywords:** Chronic obstructive pulmonary disease, Hypoxia, Hypoxia-inducible factor, Exercise

## Abstract

**Background:**

Chronic Obstructive Pulmonary Disease (COPD) is a chronic, noncommunicable disease characterized by hypoxemia, with altered lung function, dyspnea on mild exertion, limited tolerance to physical exertion, and functional impairment. Physical exercise has been recommended worldwide as an efficient strategy to improve the autonomy and quality of life of patients affected by COPD. However, the adaptive molecular mechanisms occurring in these patients after the exposure to the hypoxic stimulus triggered by physical exercise have currently not been described in populations living at high altitude.

**Methods:**

The clinical trial we are presenting here consists of a quasi-experimental design with longitudinal analysis of repeated measures, with *intra-* and *inter*-*group* comparisons, measuring primary and secondary variables in 4 temporal points. Participants will be people with a diagnosis of COPD residing at high altitudes (> 2600 m), without oncological, renal, cardiac, or musculoskeletal comorbidities with a low level of physical activity. The intervention will be an 8-week program of physical exercise of resistance and muscular strength (8-WVP) which will be carried out at home. Primary outcome variables will be the expression of HIF-1α, VEGF, and EPO. As secondary outcome variables, we will consider lung function (measured by spirometry), physical performance (measured by ergospirometry and dynamometry), and hematological parameters.

**Discussion:**

The results obtained after the clinical trial proposed here will promote knowledge on the expression of signaling proteins as an adaptive response to hypoxia in people with COPD living at high altitude, which will be relevant because there are not data on this population group. The knowledge generated from the application of this protocol will increase the pathophysiological understanding of the disease and future medical and therapeutic decision-making based on physical exercise prescription.

**Trial registration {2a}:**

NCT04955977 [ClinicalTrials.gov]—NCT04955977 [WHO ICRTP]. First Posted: July 9, 2021.

**Supplementary Information:**

The online version contains supplementary material available at 10.1186/s13063-023-07698-y.

## Administrative information

Note: Numbers in brackets refer to item numbers presented in the SPIRIT Checklist

**Table:** 

Title {1}	Effects of a physical exercise program on HIF-1α in people with chronic obstructive pulmonary disease living at high altitude: study protocol for a clinical trial
Trial registration {2a and 2b}	ClinicalTrials.gov: NCT04955977 WHO ICRTP: NCT04955977. First Posted: July 9, 2021 [https://clinicaltrials.gov/ct2/show/NCT04955977].
Protocol version {3}	Version 1 of 2–2022.
Funding {4}	This protocol is funded by the Universidad Nacional de Colombia, through the “Call for the support of research projects and artistic creation of the Bogotá campus—2019, code HERMES, 47970”.
Author details {5a}	WVP – Universidad Nacional de Colombia; Bogotá D.C, Colombia. ECM—Universidad Nacional de Colombia; Bogotá D.C, Colombia. JRT – Universitat de Barcelona; Barcelona, Spain. EMS—Universidad Nacional de Colombia; Bogotá D.C, Colombia.
Name and contact information for the trial sponsor {5b}	Universidad Nacional de Colombia research information system: hermes@unal.edu.co
Role of sponsor {5c}	Responsible for the allocation of resources for research projects. They do not interfere in the development of the research.
Roles and responsibilities: committees {5d}	It is made up of the principal investigator, the directors of the research group, the medical group, the ethics committee, and the financing committee. They are responsible for coordinating and controlling the execution of the research according to what is accepted by the ethics committee

## Introduction

### Background and rationale {6a y 6b}

Chronic Obstructive Pulmonary Disease (COPD) is characterized by chronic airflow obstruction associated with chronic airway inflammation, lung parenchymal remodeling, and hypoxemia. The clinical symptoms with the highest incidence in people with COPD are dyspnea, limited tolerance to physical exertion, functional impairment, and impaired quality of life [1]. However, environmental aspects such as acute or chronic exposure to hypobaric hypoxia environments entail even greater detriment to the clinical condition and physical performance of the COPD patients [2–4].

Exposure to hypoxia associated with pulmonary pathological conditions, physical exertion, or a decrease in the atmospheric pressure of oxygen (PO_2_ ) leads to changes at the cellular level related to oxidative stress, an increase in reactive O_2_ species, mitochondrial autophagy, and cell apoptosis [5]. Hypoxia-inducible Factor 1 (HIF-1) is the master regulatory molecule involved in the different mechanisms resulting from a low PO_2_ exposure. HIF-1 triggers several responses to hypoxia by regulating the expression of several genes that affect protein synthesis processes related to angiogenesis, erythropoiesis, and metabolic regulation, among others [6, 7].

In sports training, exposure to hypobaric hypoxia has been used as a strategy to optimize physical performance after the activation of signaling pathways derived from the increase in HIF-1 [8]. Because physical exercise is a powerful biomolecular stimulator of the hematopoietic [9], muscular [10], and endocrine systems [11], different therapeutic physical training strategies have been developed under hypoxic conditions to improve the physiological state of people affected by diseases such as heart disease, obesity, type 2 diabetes, arterial hypertension, and coronary syndromes.

Currently, there is scientific evidence on how the pathophysiological mechanisms of COPD affect HIF-1 expression [12–14] and its relationship with the inflammatory response of the airway, chronic inflammation of the lung parenchyma [15, 16], and vascular regeneration [17], especially in smokers. However, the expression of HIF-1α in patients with respiratory disease residing in hypobaric hypoxic environments which simultaneously have the hypoxemic stimulus associated with exercise (i.e., patients exercising at high altitude), is currently unknown [6]. Thus, the quasi-experimental clinical trial presented here seeks to fill this gap by determining the responses of patients with COPD living at high altitudes after being engaged in an 8-week exercise training protocol. Several by-products triggered by HIF-1α such as angiogenesis mediated by vascular endothelial growth (VEGF) synthesis, erythropoiesis stimulated by erythropoietin (EPO), and hematological changes including hemoglobin mass (Hbt) will be determined. Moreover, physical performance-related parameters measured by ergospirometry, spirometry, dynamometry, and performance field tests will be undertaken to assess functionality.

The results obtained from this research will favor a greater and better pathophysiological understanding of COPD in patients living at high altitude. Additionally, studying the molecular changes derived from the simultaneous exposure to hypobaric hypoxia and physical exercise will set a starting point for prescribing the dosage of physical exercise as a therapeutic strategy in these patients.

### Objective {7}

The main objective of this intervention and research protocol is to determine the response of HIF-1α in patients with COPD residing at high altitude (> 2600 m) before and after the 8-Week Variation Program (8-WVP) consisting of respiratory training, resistance training, strength, flexibility, elasticity, and with a health education component. The intervention will have the following secondary objectives: (1) to establish the behavior of HIF-1α at rest and in acute response to physical exercise, (2) to determine the response of VEGF and EPO after the physical training program and its correlation with HIF-1α, (3) to identify the correlation between the expression of HIF-1α and the changes in exercise capacity in patients with COPD living at high altitude after the 8-WVP, (4) to determine the effects of the physical exercise program on fitness and the quality of life of people with COPD.

### Trial design {8}

The 8-WVP exercise program will be performed by an experimental group (EXP) and by a control group (CON) to determine the differential changes that the program would cause both in patients with COPD and in healthy people. The allocation ratio of the participants will be 1:1.

## Methods

### Study setting {9}

**Fig. 1:**
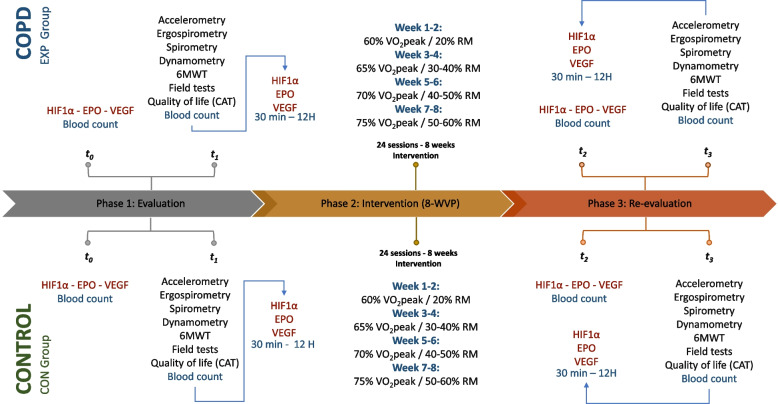
Study design. CAT: Quality Questionnaire of life in people with COPD. EPO: Erythropoietin. HIF-1α: Hypoxia-Inducible Factor 1 Alpha Subunit. t0: measurement at rest before the start of the exercise program. t1: measurement in acute and sub-acute response to physical effort before the start of the exercise program. t2: measurement at rest after the execution of the exercise program. t3: measurement in acute and sub-acute response to the physical effort after the execution of the exercise program. VEGF: Vascular Endothelial Growth Factor. V̇O_2_peak: peak oxygen consumption. 1MR: One-repetition maximum. 6MWT: 6-min walk test HIF-1α: hypoxia inducible factor 1 alpha subunit, EPO: erythropoietin, MR: maximum repetitions, VEGF: vascular endothelial growth factor, V̇O_2_peak: peak oxygen consumption

A quasi-experimental clinical trial will be carried out with a longitudinal analysis of repeated measures, with *intra-* and *inter-group* comparisons, which will have 4 measurement moments (Fig. 1): *t*_*0*_, measurement at rest before the start of the exercise program; *t*_*1*_, measurement in acute and sub-acute response to physical effort before the start of the exercise program; *t*_*2*_, measurement at rest after the execution of the exercise program; and *t*_*3*_, measurement in acute and sub-acute response to the physical effort after the execution of the exercise program. The measurements will be made at the Exercise Physiology Laboratory of the Faculty of Medicine of Universidad Nacional de Colombia. The approach to the study protocol has been developed following the guidelines for Standard Protocol Items: Recommendations for Interventional Trials (SPIRIT) [18] (Appendix 1). A flow SPIRIT diagram is shown in Table 1.

**Table 1:** Summary of the enrolment, interventions, and assessments – SPIRIT Diagram

Timepoint	Allocation	Baseline		8-WVP (8 weeks)	Close-out	
	− *t*_1_	*t*_0_	*t*_1_		*t*_2_	*t*_3_
**Enrolment:**						
Eligibility screen	X					
Informed consent	X					
Medical history	X					
Physical activity level	X					
**Interventions:**						
EXP group				X		
CON group				X		
**Assessment:**						
Protein quantification HIF-1α		X	X		X	X
Protein quantification VEGF		X	X		X	X
Protein quantification EPO		X	X		X	X
Ergospirometry		X			X	
Spirometry		X			X	
Dynamometry		X			X	
6MWT		X			X	
Field tests		X			X	
Quality of life (CAT)		X			X	
Blood count		X			X	
**Safety monitoring:**						
Vital signs		x	x		x	x
Adverse event reporting			x		x	X

### Eligibility criteria {10}

Patients with COPD will be recruited for the experimental group (EXP) and people without respiratory disease for the control group (CON). The following inclusion criteria for COPD patients will be considered: (1) to be over 60 years old residing at high altitude (> 2600 m), (2) to have pulse oximetry measures of peripheral arterial oxygen saturation (SpO_2_) at rest below 90%, (3) to not require home oxygen therapy, and (4) to have not a record of respiratory exacerbations or hospitalization in the last 3 months before the initial measurement. All patients will be selected from the General System of Social Security in Health and must sign an informed consent.

The following exclusion criteria will be considered: (1) to have been diagnosed of anemia, oncological, liver, kidney, rheumatological disease, or a history of digestive bleeding in the previous 2 months before the initial measurement, (2) to have clinical history of kidney, liver or bone marrow transplantation, (3) to be under hormonal pharmacological management with erythropoietin and/or testosterone, (4) to have acute musculoskeletal injuries that limit the execution of the physical exercise program, (5) to be an active smoker, (6) to participate in a program of pulmonary rehabilitation or in any other medical or therapeutic research, and (7) to have a moderate or high level of physical activity with a record of ≥ 600 METs/week or 1.5 METs/h [19], a weekly number of steps involving > 7000 steps/day or > 100 steps/min [20], or ≥ 720 kcal/week, measured by accelerometry [21].

### Eligibility criteria for controls

The following inclusion criteria for CON group will be considered: (1) to be over 60 years old residing at high altitude (> 2600 m), (2) to be non-smoker and do not have a history of cigarette smoking, (3) to have not a record of hospitalization in the last 3 months before the initial measurement. All patients will be selected from the General System of Social Security in Health and must sign an informed consent.

The following exclusion criteria will be considered: (1) to have been diagnosed of anemia, oncological diseases, liver disease, chronic kidney disease, rheumatic diseases, history of gastrointestinal bleeding anemia, history of kidney, liver, or bone marrow transplantation, (2) to be under hormonal pharmacological management with erythropoietin and/or testosterone, (3) to have acute musculoskeletal injuries that limit the execution of the physical exercise program, (4) to perform a high level of physical activity with a record of ≥ 600 METs/week or 1.5 METs/h, a weekly number of steps involving > 7000 steps/day or > 100 steps/min, or ≥ 720 kcal/week.

Aspects related to age, sex, body composition, vital signs, and level of physical activity will not present significant differences, in order to avoid bias in functional results and make comparisons between the study groups.

## Interventions

### Intervention description {11a}

**Fig. 2:**
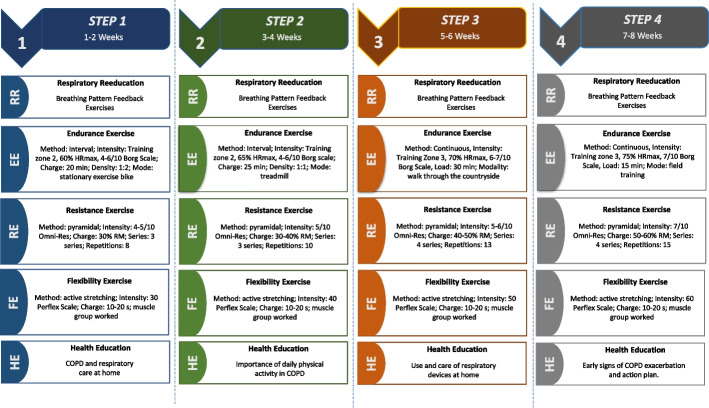
Physical exercise program RR: respiratory reeducation, EE: endurance exercise, RE: resistance exercise, FE: flexibility exercises, HE: health education, HRmax: maximum heart rate, OMNI-RES Scale: perceived effort scale in strength exercises, RM: maximum repetition, PERFLEX Scale: scale of perceived exertion in flexibility exercises, COPD: chronic obstructive pulmonary disease

The program will consist of 8 weeks with 3 sessions per week of physical exercise, for a total of 24 sessions. Each week will include sessions for training the conditional physical capabilities and sessions for health education. The training loads will be adjusted from the increased volume of work, the intensity of the exercise, and the training method. The program presents the following specifications (Fig. 2).

### Explanation for the choice of comparators {6b}

The CON group will perform the same 8-WVP exercise program as the EXP group, since the purposes of the investigation are to determine the differential molecular and physiological responses between people with COPD and people of CON.

### Criteria for discontinuing or modifying the interventions {11b}

Study participants may withdraw from the study at any time during the protocol. If adverse events associated with the intervention or the measurements occur concurrently, the study will end and the data obtained up to that moment will be analyzed. The following symptoms will be considered as discontinuation criteria: coughing, bronchospasm, chest pain, dizziness, headache, and exacerbation signs of respiratory distress after finishing the physical effort.

If an uncontrolled exacerbation of respiratory symptoms occurs after the physical exercise program, non-pharmacological management will be given as follows: (i) start oxygen therapy until a SpO_2_ level ≥ 90%, (ii) maneuvers of feedback of the respiratory cycle, (iii) constant monitoring of respiratory pattern and vital signs, (iv) accompaniment to the nearest health center. If it would be necessary to start respiratory pharmacological treatment, the specific medication to each patient according to their personal medical formulation will be administered.

### Strategies to improve adherence to interventions {11c}

As part of the intervention for the 8-WVP program, health education training and workshops will be developed regarding respiratory and musculoskeletal care topics during and after the practice of physical exercise, as well as topics associated with the benefits of physical exercise. This education training is aimed to promote the understanding of the participants in the 8-WVP program to improve their adherence to the study.

#### Patient participation in study design

The participants in this research will decide the specific concepts to be developed in the education sessions, considering the particular needs of each subject, the tastes and preferences of knowledge, and the thematic areas proposed in Fig. 2. The identification of the concepts to be developed will be done through open questions during the development of the physical exercise sessions.

### Relevant concomitant care permitted or prohibited during the trial {11d}

Participation in alternate physical activity or physical exercise programs implemented by other professionals. Pharmacological use of erythropoietin (EPO), corticosteroids, nonsteroidal anti-inflammatory, and antibiotics.

### Provisions for post-trial care {30}

A telephone follow-up for 3 weeks after participation in the study will be tracked to assess complications after the study and to continue the promotion of healthy lifestyle habits. Once the study will be over, the results will be communicated to all the participants in a joint session.

### Outcomes {12}

The primary outcomes of this investigation will be related to the expression of the HIF-1α protein and its incidence on the response of VEGF-mediated angiogenesis and EPO-regulated erythropoiesis. Secondary outcomes will be associated with the performance of lung function (measured by spirometry), physical performance (measured by ergospirometry and dynamometry), and hematological changes after physical exercise (measured by systematized complete blood count).

#### Primary outcomes

–Protein expression by enzyme-linked immunosorbent assay (ELISA). To analyze of the primary variables HIF-1α, VEGF, and EPO, 8 mL of venous blood will be taken. Blood will be centrifuged at 2400* g* for 10 min at 4 °C to isolate the plasma, which will be separated into aliquots of 200 μL in Eppendorf microtubes and subsequently stored at − 80 °C in the Exercise Physiology Laboratory of the Universidad Nacional de Colombia until analysis. Protein quantification HIF-1 [22], VEGF [23], and EPO [24] in plasma will be carried out using the sandwich-type ELISA molecular technique using specific antibodies to immobilize the study proteins on the plate for their subsequent detection. Finally, a chemiluminescence detection will be performed using a spectrophotometer SpectraMax ®M5 (San José, USA). The following antibodies will be used: (i) human/mouse total HIF-1α DuoSet – R&D Systems, (ii) human VEGF quantikine ELISA Kit – R&D Systems, (iii) human erythropoietin quantikine—R&D Systems (Minneapolis, USA).

#### Secondary outcomes

- Pulmonary function. It will be evaluated by means of spirometry, employing spirometer COSMED microQuark PC (Rome, Italy) and using the Omnia 2.0 COSMED operating system (Rome, Italy). The execution procedure and analysis of the results will be carried out following the recommendations of the American Thoracic Society (ATS) and European Respiratory Society (ERS) [25]. The valid measures will be considered after the highest value of three satisfactory measurements. The parameters to analyze will be Forced Expired Volume in the first second (FEV1), Forced Vital Capacity (FVC), FEV1/FVC Ratio, and changes in FEV1 (ΔFEV1) and FVC (ΔFVC).
- Ergospirometry. Cardiopulmonary exercise testing (CPET) will be performed using a Velotron DynaFit Pro cycle ergometer—RacerMate (Seattle, USA) adjusted for the comfort of the participants. The Cosmed Quark-B2 gas analysis system (Rome, Italy) will be used to measure the variables resulting from the cardiopulmonary test. An incremental protocol will be followed by all participants, respecting the physical tolerance of each participant [26]. The protocol will consist of (1) 1 min at rest on the cycle ergometer, (2) 2 min of warm-up at 30 rpm without load, (3) beginning of the test with a pedaling at 60 rpm for 2 min with a load of 30 W and increasing 15 W the load every 2 min until fatigue, (4) 2 min of active recovery, pedaling without load at 40 rpm, (5) 4 min of passive recovery in a sitting position. The following parameters will be analyzed: maximum oxygen consumption (V̇O_2_max), peak oxygen consumption (V̇O_2_peak), carbon dioxide production (V̇CO_2_), pulse-oximetry (SpO_2_), maximum voluntary ventilation (MVV), ventilatory reserve (VR) (calculated after 1 min of maximal exercise ventilation: V̇Epeak/MVV), maximum heart rate (HR max), heart rate reserve (HRR) (calculated as heart rate at maximum exercise: HR peak/HRmax) [27].
- Muscular strength. The measurement of peripheral muscle strength will be carried out using a digital dynamometer (Saehan—model DHD-1, Seoul, Korea) and performing the test on the dominant limb. Three repetitions with 1 min of rest between them will be recorded and the highest value will be considered [28].
- Automated blood count. Venous blood collection will be carried out from a peripheral blood sample by venous puncture in the antecubital vein. Before the procedure, a tourniquet will be made and a 21-gauge needle in a 10-cc hypodermic syringe will be used. Blood will be collected with the Vacutainer collection system and the sample placed in tubes with ethylenediaminetetraacetic acid (EDTA). For each participant, 4 mL of venous blood will be collected for an automated blood count in Mindray CAL 6000 Blood Cell Analysis System (Shenzhen, China). The following parameters will be obtained: reticulocyte count, red blood cell (RBC) count, hemoglobin concentration ([Hb]), hematocrit (Hct), mean corpuscular volume (MCV), mean corpuscular hemoglobin (MCH), mean corpuscular hemoglobin concentration (MCH).
- Physical activity level. The level of physical activity will be measured using an ActiGraph GT3X + triaxial accelerometer (Pensacola, USA) which registers acceleration with variations of magnitudes from 0.05 to 2.5 g (g = 9.8 m/s^2^) [29] with a frequency range of 0.25 to 2.5 Hz for 60 s. The accelerometer will be attached to the participant’s dominant waist or hip using a belt. Recordings will be obtained during 7 days, taking as an efficient measurement of 5 valid days for which the first and last day of measurement will be excluded [30]. A period of continuous use of the accelerometer with time from 8 to 10 h will be taken as a valid day. For the analysis of the data, the times of non-registration will be excluded, defined as the period of 60 min of counting 0, which is the evidence of non-use of the device. To define the level of physical activity, the records are as follows: (i) METs/h, METs/day and average METs, (ii) energy expenditure in kcal/h, kcal/day and average kcal, (iii) number of steps per hour, number of steps per day, and average number of steps.
- Quality of life associated with health. To evaluate quality of life, the COPD Assessment Test (CAT) will be applied, which is a survey that determines the person’s quality of life in relation to the impact of the disease on the patient’s well-being. Comparisons of health-related quality of life (CAT) will be performed exclusively in the COPD group to determine the effects of the physical exercise program in subjects with the same health condition.

### Participant timeline {13}

**Fig. 3:**
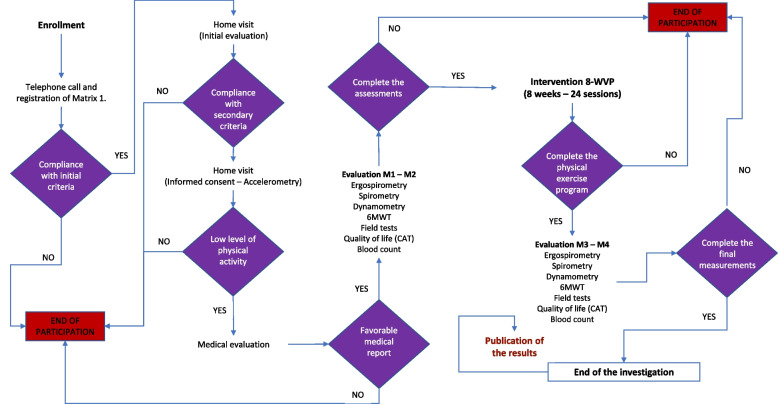
Chronology of enrollment, interventions, and evaluations

The enrollment procedure, interventions, and evaluations of the participants are presented below (Fig. 3):

- Day 0: Selection and enrollment. Snowball recruiting.
- Day 1. Determination of health condition: (1) cardiovascular health condition with electrocardiogram, (2) identification of neurological or musculoskeletal comorbidities, (3) filling in the clinical history of the investigation, and (4) signature of informed consent.
- Day 2. Evaluation of physical performance: (1) lung function by spirometry, (2) measurement of cardiopulmonary resistance by ergospirometry, (3) assessment of muscle strength in extremities using dynamometry, (4) systematized blood count, (5) ELISA determination, and (6) quality of life assessment with CAT questionnaire in people with COPD.
- Day 3 to day 27. Each participant will follow the 8-WVP program at home.
- Days 28 and 29. Reassessment of physical performance through the same tests and protocols developed on day 02 and discharge evaluation.
- Day 30. Socialization of the results to participants and interested parties.

### Sample size {14}

The sample size for the longitudinal studies was calculated using the Diggle formula [31] for both differences between individuals and between groups, taking as reference an expected effect size of 7.10 (*δ*) for the expression of EPO, VEGF, and HIF-1α. Variances (*δ*2) of 6.76 are considered according to each effect measure reported in the literature [14, 32, 33]. Two measurements will be considered (pre and post) for the two comparison groups, with a type I error of *α* = 0.05, power *P* = 0.80 and exploring correlations (*ρ*) of 0.47 according to each parameter. In this way, a total of 10 people are projected and an attrition of 20% is calculated. Therefore, 6 people with COPD are projected for the experimental group and 6 people without COPD for the control group. Being a study of repeated samples, a total of 6 measurements per participant will be analyzed for a total of 72 samples for the analysis of the results.

### Recruitment {15}

The recruitment process will be carried out by snowball involving people attending the National University hospital - UN and the Universidad Nacional de Colombia. The principal investigator will contact the interested participants by a telephone call in which the nature of the study will be explained. If people express interest in participating, a home visit will be scheduled to explain the details of the study, resolve concerns, and provide any necessary clarifications. After confirmation of compliance with the selection criteria, the informed consent will be signed, and then the evaluation process will start. Any participant who wishes to withdraw voluntarily during the study may do so and this will not affect the quality of care for the other participants.

## Assignment of interventions: allocation

### Sequence generation {16a}

The assignment of research groups will be done by convenience, to compare the effects of physical exercise on outcomes in people with COPD (EXP) versus healthy people (CON) with similar physical and sociodemographic conditions related to age, biological sex, nationality, socioeconomic level, living at high altitude, social and health security.

### Concealment mechanism {16b}

Since it will be a quasi-experimental study and the group assignment will be done for convenience, it will not apply any hidden assignment.

### Implementation {16c}

Convenience allocation will be made by the principal investigator. The recruitment of participants will be carried out by all the authors of the investigation. The 8-WVP intervention program will be followed by both groups and will be disclosed at the start of the investigation.

## Assignment of interventions: blinding

### Who will be blinded {17a}

The participants will not know in which intervention they will participate and neither they will know in which intervention the other participants will be enrolled, since the 8-WVP exercise program will be carried out individually and at each participant’s home.

### Procedure for unblinding if needed {17b}

Due to the design of the protocol, there is no unmasking process.

## Data collection and management

### Plans for assessment and collection of outcomes {18a}

The data of the participants will be obtained from the initial interview and medical evaluation. The data will be compiled in a Clinical History form created for this research. The laboratory and physical stress tests will be performed in the Exercise Physiology Laboratory of the Faculty of Medicine of the Universidad Nacional de Colombia and the molecular analyses will be carried out at the Laboratory of Basic Research in Biochemistry of the Universidad Nacional de Colombia.

### Plans to promote participant retention and complete follow-up {18b}

Before the start of the research, meetings with the participants will be held to explain the details of the study, the requirements for their participation, and the commitments to be acquired. During the measurements and physical exercise sessions, the attendance control will be registered in a format specifically designed for this research. Each participant will perform the training sessions at home, which will reduce the desertion of the participants.

### Data management {19}

The data will be processed by the researchers to protect the identity of each participant through the use of randomly assigned codes using the Oxford Minimization and Randomization (OXMar) free software, which is an efficient randomization, concealment, and minimization system for clinical studies with an adaptation to Spanish language (36).

The digital information resulting from the investigation will be stored by the principal investigator on two USB sticks for 2 years after the end of the investigation. Each of the documents will be protected using the Encryption technique with a secret key, which will be known exclusively by the researchers.

To guarantee the quality of the information on the physical formats, audits will be carried out to review the correction of the completed forms. For this procedure, two data matrices will be built separately in the Epidata version 3.1 software. Additionally, a validation of the data will be carried out to correct typing errors employing the “Validate” subprogram of the Epidata version 3.1 software. In the construction of the data matrices, the “Check” subprogram of Epidata version 3.1 will be used to restrict data capture and thus reducing typing or coding errors.

The signed informed consent forms will be filed in an AZ folder and kept in a locked drawer in the medical school exercise physiology laboratory of the Universidad Nacional de Colombia Department of Human Body Movement. The researchers will in no way reveal the data of the participants to any source. The data obtained will be used exclusively for academic and research purposes.

## Statistical methods

### Statistical methods for primary and secondary outcomes {20a}

#### Descriptive analysis

The variables in the qualitative measurement scale will be described as proportion frequencies, and the continuous variables will be described with measures of central tendency (mean and median) and dispersion (standard deviation, interquartile range) according to whether or not they have a normal distribution. Data will be presented with graphic (histograms, dispersion) and numerical methods (tables).

#### Baseline analysis

To compare the two groups, parametric and non-parametric tests will be performed according to the verification of a priori criteria. For comparisons between proportions, chi-square or Fisher’s exact will be used, and for comparison of continuous variables between groups, ANOVA or Kruskal–Wallis tests will be run.

#### Multivariate analysis

As a longitudinal method, a repeated measures analysis will be carried out for correlated data. For each of the continuous outcome variables, the analysis will be performed with (marginal) fixed effects models. The evaluation of the assumption of normal distribution of the variables will be carried out using graphic methods (histograms, box plots, and QQ graphs).

For variables with normal distribution, the restrictive maximum likelihood model will be used to select the best correlation matrix. Subsequently, to select the covariates to keep in the model, the maximum likelihood estimate will be used. The selection of the best model will be based on the lowest value of the Akaike Information Criterion and the Bayesian Evaluation Criterion after having evaluated the interactions and non-linear terms. For conditional models, tests with intercepts and slopes will be used. If there is no normal distribution, a generalized equation estimation model with an unstructured matrix will be implemented. A residual analysis will be performed on all final models. All the statistical approach described above will make possible to evaluate the possible effects both within and between groups with a robust model analyzing the outcome measures and control for possible confounding variables.

### Interim analyses {21b}

Interim analyses are not planned.

### Methods for additional analyses (e.g., subgroup analyses) {20b}

No further subgroup analyses are planned.

### Methods in analysis to handle protocol non-adherence and any statistical methods to handle missing data {20c}

Primary and secondary outcomes will be performed using an intention-to-treat analysis. Missing data will be minimized using the strategies listed above.

### Data access and plans to give access to the full protocol, participant-level data, and statistical code {29 and 31c}

All data and annexes related to this study will be available upon request to the principal investigator subject to the intellectual property guidelines of the funding institution.

## Oversight and monitoring

### Composition of the coordinating center and data monitoring committee {5d and 21a}

This research will be carried out, coordinated, and controlled by the research group on adaptations to exercise and hypoxia of the Universidad Nacional de Colombia. The development of this study is developed by:

- Principal investigator: Physiotherapist, specialist in cardiorespiratory physiotherapy, MSc in Sports and physical activity physiotherapy, and Ph.D. in science - biology. Will perform the measurements and interventions of all the participants linked to the study.
- Director and Co-director of the study: The director is a Biologist with a Ph.D. in physiology. The co-director is a Physiotherapist with a Ph.D. in science-biology. Will carry out the orientation of conceptual issues and logistical aspects for the development of the tests, as well as give orientation to events related to the execution.
- Medical group of the study: It will be made up of a sports doctor and two internal medicine doctors. Will accompany the process of evaluating the physical aptitude of the participants and will inform them about the health condition of the participants during the recording of the measurements.
- Daily monitoring group: It will be made up of a physiotherapist and a nurse. They will monitor the clinical evaluation and complications present during the exercise sessions.

### Adverse event reporting and harms {22}

All adverse events reported by the participants or seen by the investigators will be recorded in the Clinical Record of each participant. According to the type of the adverse event, it will be decided to carry out the appropriate medical or physiotherapeutic treatment, and a follow-up will be undertaken until resolution.

### Frequency and plans for auditing trial conduct {23}

A committee of experts in the field has been constituted for the initial evaluation of this study. This committee is composed by a physician from the Federal University of São Paulo—Brazil), a physician from the University of Sabana – Colombia, and a biologist from the Universidad Nacional de Colombia (Certificate 16 Mar 2021—Biology Curricular Direction). These experts decided that the study is safe and does not pose any risk to the participants’ health or well-being. The adequate progress of the project will be assessed every 4 months by the Director of the research group to which this project is subscribed. Finally, audits by authorities under the Universidad Nacional de Colombia or the Ministry of Science, Technology, and Innovation of Colombia will be carried out.

### Plans for communicating important protocol amendments to relevant parties (e.g., trial participants, ethical committees) {25}

Potential substantial modifications of the protocol will be reported to the Biology postgraduate Advisory Committee and to the Ethics Committee of the Faculty of Sciences of the Universidad Nacional de Colombia, who authorized the execution of the protocol.

### Dissemination plans {31a}

All the results of this research will be disclosed in international scientific journals with a peer review policy. Likewise, it is planned to make disclosures in academic events with scientific committees. At the end of the investigation, the results will be disseminated to the participants and their families.

## Ethics and dissemination

### Research ethics approval {24}

This research has the endorsement of the ethics committee of the Faculty of Sciences of Universidad Nacional de Colombia, granted on December 7, 2020, and registered in act 14–2020 (accreditation of the ethics committee, resolution No. 023 OF 2020, act 08 of 047/16/2020). Additionally, it has the approval of the research committee in which 3 international peer reviewers (a pulmonologist, a sports doctor and a molecular biologist) determined the relevance and scientific rigor of the protocol (act 015 of May 20, 2021—Council Resolution 0310). This protocol is registered in https://clinicaltrials.gov/ with number NCT04955977.

### Who will take informed consent? {26a}

All participants who meet the eligibility criteria of this research will sign the informed consent, either from the EXP or CNT group. After the evaluation and medical approval, the specific aspects of the research will be explained to each person and the informed consent will be signed.

### Additional consent provisions for collection and use of participant data and biological specimens in ancillary studies {26b}

No further studies are planned with the participant data.

### Confidentiality {27}

During the development and at the end of this research, the personal data of all participants will be protected. The data collected will be processed exclusively by the principal investigator using alphanumeric coding for each of the participants.

### Plans for collection, laboratory evaluation, and storage of biological specimens for genetic or molecular analysis in this trial/future use {33}

The biological samples will be stored frozen at − 80 °C until their analysis in the Exercise Physiology Laboratory and the Basic Research Laboratory in Biochemistry of the faculty of science of Universidad Nacional de Colombia according to the protocols established by the institution.

### Declaration of interests {28}

None of the authors have a conflict of interest.

### Consent for publication {32}

This manuscript does not contain the individual personal data of patients.

## Discussion

### Potential impact and importance of the study

Despite the great worldwide interest in the knowledge of the adaptive changes to hypoxia in areas such as sport and the knowledge of the physiological effects of altitude on the transcription of HIF-1α and its role in the adaptation to chronic hypoxemia of chronic noncommunicable diseases [2], it is not yet known what is the effect of physical exercise on the adaptive molecular changes to hypoxia in people with COPD living at high altitude and how these adaptations would improve physical performance and fatigue tolerance. It is hypothesized that the application of this physical exercise protocol will increase the expression of HIF-1α, which would be related to an increase in the synthesis of VEGF and EPO. This sequence of events would improve physical performance in tests of physical aptitude and muscle functionality. In this way, the application of this protocol will increase the knowledge of the molecular mechanisms of adaptation to hypoxia caused by exercise in people with COPD and will add some clues for promoting physical exercise programs in these patients according to their clinical condition and the degree of environmental hypoxia in which they reside.

### Strengths and limitations of the study

The strengths of this study are as follows: (i) it is the first study to simultaneously evaluate the impact of environmental hypoxia and hypoxemia generated by physical exercise in people with COPD living at high altitude, regarding the mechanisms of action of HIF-1α, (ii) it will measure the adaptive responses associated with VEGF angiogenesis and EPO both at rest and after acute physical exercise, (iii) it will identify the correlation between hematological variables and physical performance from cardiopulmonary stress tests in a poor studied population group, (iv) it will involve the translation of molecular results to clinical research derived from functional tests. These measures are objective and will strengthen the current knowledge on COPD and its treatment in high altitude residents.

The fact that we do not have a control group at sea level is considered a limitation of this protocol; however, the results of this study will be the starting point for the execution of investigations with the same characteristics at different altitudes.

### Contribution and clinical applicability

Articles have recently been published relating to the effects of exercise programs on people with COPD [34]. However, and to the best of our knowledge, there are no data on this type of patient at high altitudes; therefore, the biological and physiological knowledge of people with this COPD who live in environments with low PaO2 is scarce and does not take into account the hypoxic stimuli that we intend to study.

The results obtained in this research will promote knowledge of the molecular responses concerning protein synthesis as an adaptive response to hypoxia in people with COPD, which will have an important impact on the understanding of the pathophysiology of the disease and decision-making. Future medical and therapeutic decisions based on physical exercise will benefit from the results derived from the study. This research is articulated with the WHO/PAHO development plan to combat the incidence of COPD in the Americas, which seeks to improve the response of health systems against chronic noncommunicable diseases.

## Trial status

The recruitment period began on August 22, 2022. In the calling phase, a total of 45 people with a medical diagnosis of COPD interested in participating in the study were registered. The protocol is version 1–2022.

### Supplementary Information

**Additional file 1. **

## Data Availability

All data and annexes related to this study will be available upon request to the principal investigator subject to the intellectual property guidelines of the funding institution.

## Abbreviations

CAT: Quality Questionnaire of life in people with COPD
COPD: Chronic obstructive pulmonary disease
CPET: Cardiopulmonary exercise testing
EPO: Erythropoietin
FEV1: Forced Expired Volume in the first second
FEV1/FVC: Ratio Forced Expired Volume in the first second and Forced Vital Capacity
FVC: Forced Vital Capacity
Hb: Hemoglobin
Hct: Hematocrit
HIF-1α: Hypoxia-Inducible Factor 1 Alpha Subunit
MCV: Mean corpuscular volume
MCH: Mean corpuscular hemoglobin
MR: One-repetition maximum
MVV: Maximum voluntary ventilation
RBC: Red blood cell
HRmax: Maximum heart
HRR: Heart rate reserve
PaO_2_: Arterial partial pressure of oxygen
SpO_2_: Pulse oximetry
VEGF: Vascular endothelial growth factor
V̇CO_2_: Carbon dioxide production
V̇O_2_max: Maximum oxygen consumption
V̇O_2_peak: Peak oxygen consumption
VR: Ventilatory reserve
6MWT: 6-Min walk test
